# The Beat

**Published:** 2010-11

**Authors:** Erin E. Dooley

## EFSA on Revising BPA Guidance: Not Enough Evidence

In September 2010 the European Food Safety Authority (EFSA) released the findings of its latest review of bisphenol A (BPA), concluding there is no new evidence that warrants a revision of the current Tolerable Daily Intake of 0.05 mg/kg body weight.^1^ EFSA also concluded that currently available animal data do not provide convincing evidence of neurobehavioral toxicity of BPA. The EFSA panel said it would reconsider the current opinion should new relevant data become available.

## EPA Issues SNURs for Carbon Nanotubes

Significant new use rules went into effect 18 October 2010 for generic multi-walled carbon nanotubes and single-walled carbon nanotubes.^2^ Carbon nanotubes currently are used in applications such as advanced composites, electronics, and fuel cells. Now companies that manufacture, import, or process these materials must notify the U.S. EPA 90 days before using them in a way that is deemed a significant new use. In May the GAO issued a report calling on the EPA to strengthen its oversight of nanomaterials used in commerce.^3^

**Figure:**
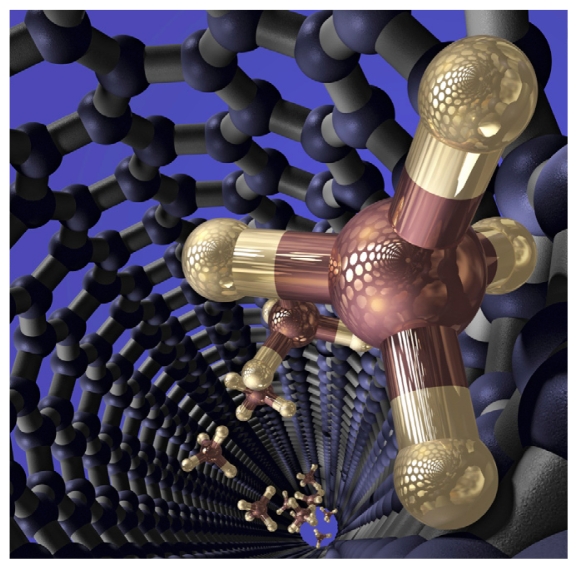


## PM Pollution: An App for That

University of Southern California researchers have developed a smartphone application to estimate atmospheric particulate matter.^4^ The app currently works with Android systems, and an iPhone app is being developed. Users upload their photographs of the sky to a central computer, which compares the picture with established models of sky luminance to determine visibility, a measure associated with particulate pollution. The system then returns a message to the user and registers the information.

## Updated Green Guides Open for Comment

In June 2010, *EHP* reported on the growing use of environmental stewardship claims in product marketing.^5^ Now the Federal Trade Commission has issued proposed changes to its Green Guides, which aim to help marketers determine if their “green” claims are true and substantiated.^6^ The Green Guides were last updated in 1998, well before a recent escalation in the number of advertisements touting claims of environmental friendliness.^5^ The proposed revisions include new guidance on the use of product certifications and other labeling tools. They also contain the first federal guidelines for the marketing of carbon offsets and renewable energy claims. The proposals are open for public comment until 10 December 2010.^7^

**Figure:**
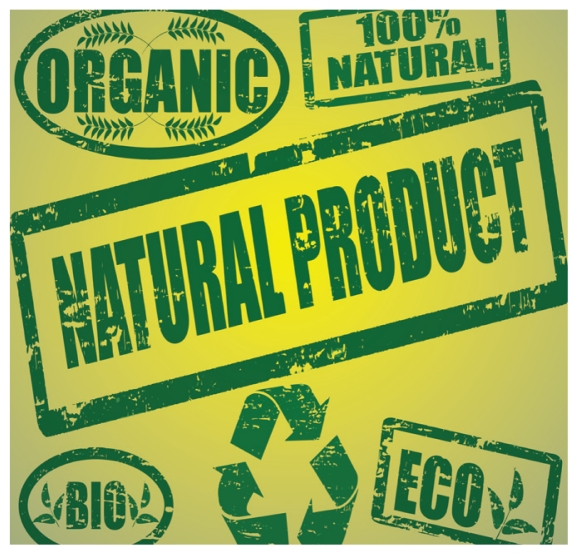


## Database of Bedbug Resources

A new online resource offered by the U.S. EPA aids consumers battling bedbug infestations.^8^ The database lists about 300 pesticides that have been registered for use on bedbugs, and users can search for products that meet specific needs. The site emphasizes the importance of proper use of pesticides. The EPA Office of Pesticide Programs advises that pesticides work most effectively against bedbugs when used along with other steps such as reducing household clutter, using protective covers on mattresses, and vacuuming regularly. Bedbugs are classified by the U.S. EPA as “a pest of significant public health importance” under the Federal Insecticide, Fungicide, and Rodenticide Act.^9^

**Figure:**
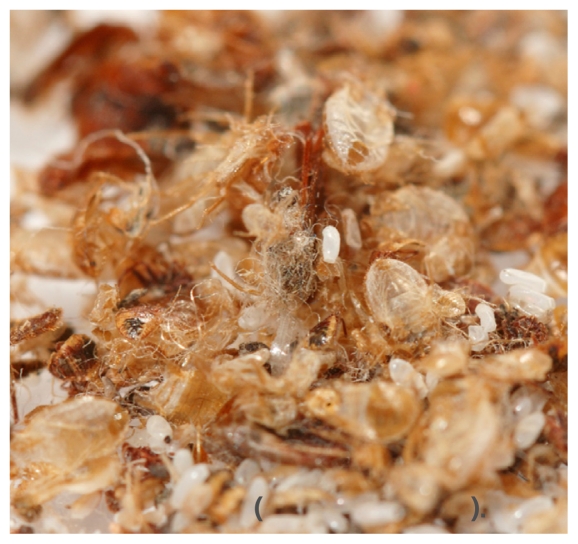
Spent skins, eggs, and carcasses of the bedbug (*Cimex lectularius*).
